# TG‐51 reference dosimetry for the Halcyon™: A clinical experience

**DOI:** 10.1002/acm2.12349

**Published:** 2018-05-21

**Authors:** Samantha A.M. Lloyd, Tze Yee Lim, Xenia Fave, Everardo Flores‐Martinez, Todd F. Atwood, Vitali Moiseenko

**Affiliations:** ^1^ Department of Radiation Medicine and Applied Sciences University of California, San Diego La Jolla CA USA

**Keywords:** Halcyon, reference dosimetry

## Abstract

Halcyon™ is a single‐energy (6 MV‐FFF), bore‐enclosed linear accelerator. Patient setup is performed by first aligning to external lasers mounted to the front of the bore, and then loading to isocenter through pre‐defined couch shifts. There is no light field, optical distance indicator or front pointer mechanism, so positioning is verified through MV imaging with kV imaging scheduled to become available in the future. TG‐51 reference dosimetry was successfully performed for Halcyon™ in this imaging‐based setup paradigm. The beam quality conversion factor, *k*_Q_, was determined by measuring %dd(10)_x_ three ways: (a) using a Farmer chamber with lead filtering, (b) using a Farmer chamber without lead filtering, and (c) using a PinPoint chamber without lead filtering. Values of *k*_Q_ were determined to be 0.995, 0.996, and 0.996 by each measurement technique, respectively. Halcyon™'s 6 MV‐FFF beam was found to be broader than other FFF beams produced by Varian accelerators, and profile measurements at *d*_max_ showed the beam to vary less than 0.5% over the dimensions of our Farmer chamber's active volume. Reference dosimetry can be performed for the Halcyon™ accelerator simply, without specialized equipment or lead filtering with minimal dosimetric impact. This simplicity will prove advantageous in clinics with limited resources or physics support.

## 1 INTRODUCTION

Halcyon™ (Varian Medical Systems, Inc., Palo Alto, CA) is a single‐energy linear accelerator introduced in North America in mid‐2017. The 6 MV‐flattening filter free (FFF) source is mounted opposite an imager and beam‐stop stack, and the gantry is enclosed in a carbon fiber bore. Because the gantry is enclosed by the bore, patient setup on Halcyon™ is performed by first aligning to external lasers mounted on the front of the bore, and then loading to isocenter through pre‐defined couch shifts. Positioning is verified or adjusted using MV imaging: either orthogonal MV image pairs, or MV cone beam computed tomography (CBCT). Performing reference dosimetry for the Halcyon™ differs logistically from other linear accelerators due to its unique features.

The American Association of Physicists in Medicine (AAPM) Task Group 51 (TG‐51) protocol is a global standard for clinical reference dosimetry of medical linear accelerators.^1^ The TG‐51 protocol for reference dosimetry is flexible when it comes to calibration conditions: source‐to‐axis distance (SAD) or source‐to‐surface distance (SSD) setup, maximum dose depth (*d*_max_) or a user‐defined depth. In contrast, the protocol is stringent in terms of measurement conditions for specification of the beam quality conversion factor (*k*_Q_). These conditions are 100‐cm SSD and 10 × 10 cm^2^ field size, therefore this protocol cannot be directly applied to machines incapable of fulfilling these conditions, such as Tomotherapy or CyberKnife. Technically, the Halcyon™ accelerator is TG‐51 compliant, as it is able to create 10 × 10 cm^2^ at 100‐cm SSD, however, typical setup with the aid of light‐projected crosshairs and front‐pointer positioning tools is not possible, posing setup challenges unique to Halcyon™. Therefore, positioning verification of both the water‐tank and stage for the Imaging and Radiation Oncology Core (IROC) optically stimulated luminescent detector (OSLD) irradiation must be performed entirely with megavoltage (MV) imaging as kV imaging is not currently available.

Use of FFF beams has expanded rapidly in recent years. Halcyon™'s 6 MV‐FFF beam profile and monitor unit (MU) rate are different compared to FFF beams produced by the TrueBeam^®^ (Varian Medical Systems). Ion recombination correction factors, *P*_ion_, are larger for FFF beams compared to flattened beams^2^ in part due to the increase in dose rate, however, the Halcyon™'s 800 MU/min dose rate is closer to the 600 MU/min dose rate offered by Varian's flattened fields, than to the 1400 MU/min or greater dose rate offered in TrueBeam^®^'s FFF modes. Regardless, the recombination correction needs to be benchmarked for Halcyon™. Furthermore, use of Farmer chambers to perform measurements of FFF beams, in particular for percent depth dose (PDD) measurements, may lead to inaccuracies due to averaging over the ion chamber active volume^3^ leading to an overestimate of the PDD at 10 cm. The beam profile from Halcyon™ does not exhibit a peak as pronounced as that observed for the TrueBeam^®^ accelerator, and the use of a Farmer chamber needs to be validated.

The original TG‐51 report^1^ stipulates that for photon beams with energies 10 MV and above, PDD measurements must be performed with a 1‐mm lead foil placed approximately 30 or 50 cm from the water surface. This procedure accounts for contamination electrons that may affect the dose at *d*_max_. The measured %dd(10)_Pb_ is corrected to determine the PDD due to photons alone, %dd(10)_X_, by means of eqs. (13) and (14) provided in the report. The addendum to the TG‐51 report^4^ specifically addresses the applicability of the protocol to FFF beams and states that the energy threshold for lead filtering applies only to beams with flattening filters, and as FFF beams contain accelerator‐produced electron contamination, lead filtering and the accompanying equations should be used for all energies. However, the equations are provided for %dd(10)_Pb_ values characteristic of beam energies greater than 6 MV, with the caveat that in the case of lower energy beams, %dd(10)_Pb_ = %dd(10)_X_. Whether this condition holds true for FFF beams is not explicitly stipulated.

This note describes our clinic's experience performing TG‐51 reference dosimetry for Halcyon™.

## 2 METHODS

### 2.A Equipment

TG‐51‐compliant reference dosimetry of the Halcyon™ 6 MV‐FFF beam was performed in a water tank (1D Scanner™, Sun Nuclear Corporation, Melbourne, FL) using a 0.6‐cc waterproof Farmer chamber (PTW 30013, Freiburg, Germany) and electrometer (Fluke F35040, Fluke Biomedical, Everett, WA) calibrated by the University of Wisconsin Accredited Dosimetry Calibration Laboratory. PDD measurements were performed with the Farmer chamber, as well as a 0.015‐cc PinPoint chamber (PTW 31014, Freiburg, Germany), with corrections for the effective point of measurement. Farmer chamber measurements of the PDD were performed with and without a 1‐mm lead foil suspended 30 cm from the water surface as outlined in the TG‐51 protocol.

In order to assess whether the Farmer chamber's dimensions were appropriate for calibration of the Halcyon's 6 MV‐FFF beam, profiles were measured using an IC Profiler (Sun Nuclear) ion chamber array. The array is made up of 251 ion chambers, each with a width of 2.9 mm, spaced 5 mm apart. Relative profiles were acquired in the crossline and inline directions at isocenter with 0.9 cm buildup, inherent in the IC Profiler.

Finally, an OSLD housed in an acrylic block provided by IROC Houston Quality Assurance Center was irradiated under specified conditions and returned for independent verification of our beam calibration.

### 2.B Setup

As there are no collimating jaws, the Halcyon™ beam is shaped entirely with two independently functioning multi‐leaf collimators (MLCs). Each leaf is 1.0 cm wide projected to isocenter, and the proximal and distal MLC banks are staggered by 0.5 cm. Current commercially available Halcyon™ machines have only MV imaging capabilities, with kV imaging scheduled to be available in the near future.

**Figure 1:**
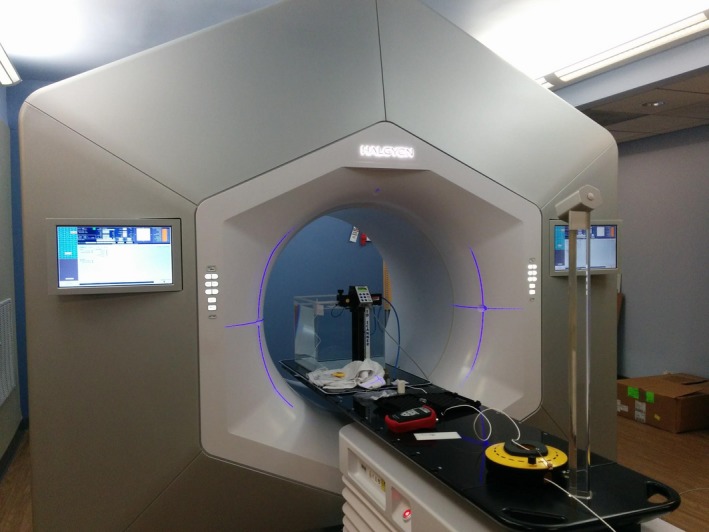
TG‐51 setup on Halcyon™.

During loading, the couch shifts from an external laser virtual isocenter to the radiation isocenter. This shift is verified through the Machine Performance Check (MPC), which must be performed daily before the Halcyon™ can be used. For the measurements performed in this work, the ion chamber was initially aligned to the virtual isocenter as indicated by the external positioning laser system, and then loaded into the bore (Fig. 1). Similarly, the IROC‐supplied stage for OSLD irradiation was initially set to the external lasers. Following loading, SSD and ion chamber positioning were fine‐tuned based on MV images acquired at gantry angles 0° and 90°. Imaging was performed in Service Mode using the “Intermediate” user profile, which is the only profile in Service Mode with access to absolute dose calibration. High‐quality MV imaging was selected from the XI tab and the number of frames acquired as well as the imaging dose were increased to improve image quality.

### 2.C Calibration procedure

The setup for Halcyon™ in the treatment planning system (TPS) and system settings allows for three possible calibration point definitions: (a) 100‐cm SSD at *d*_max_, (b) 100‐cm SAD at 5 cm depth, and (c) 100‐cm SAD at 10 cm depth. Factory settings for all Halcyon™ machines places the calibration point at *d*_max_ with 100‐cm SSD, and so this setup was chosen for our calibration condition. The factor *k*_Q_ was calculated based on PDDs for a 10 × 10 cm^2^ field obtained with both Farmer and PinPoint chambers, the former with and without the lead foil. Calculations of %dd(10)_X_ and *k*_Q_ were performed according the TG‐51 addendum and original report. Only the value of *k*_Q_ calculated using PDDs measured with the Farmer chamber was used for output calibration.

Calibration was performed using the Farmer chamber positioned at a depth of 10 cm in the water tank. The PDD curve measured with the Farmer chamber was used to correct back to *d*_max_ and the beam was calibrated to 1 cGy/MU at *d*_max_ under reference conditions (10 × 10 cm^2^ field, at 100‐cm SSD).

The IROC‐provided OSLD and stage were setup such that the stage was positioned at 100‐cm SSD and the acrylic block was placed on top of the stage with the OSLD centered along the beam axis. A 10 × 10 cm^2^ field was used to deliver 100 MU to the OSLD.

## 3 RESULTS

**Figure 2:**
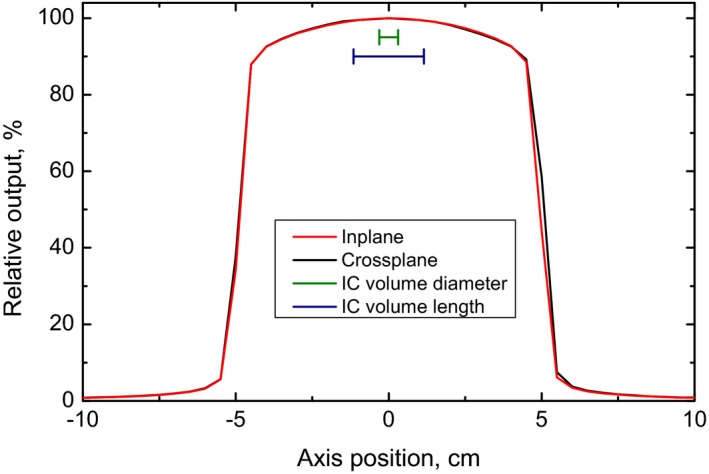
Crossplane and inplane profiles for the Halcyon™ 6 MV‐FFF beam as measured by an IC Profiler at isocenter with 0.9 cm buildup. The active volume dimensions (width and length) of the Farmer chamber used to perform calibration measurements is indicated by the green and blue bars.

Relative profiles in the crossplane and inplane directions for a 10 × 10 cm^2^ field delivered at isocenter with 0.9 cm buildup are displayed in Fig. 2. The green and blue bars indicates the diameter and length of the Farmer chamber's active volume. Within this part of the profile, the variation in dose in both directions is less than 0.5%, and so the Farmer chamber was deemed appropriate for calibration of the beam.

### 3.A Determination of *k*_Q_

**Figure 3:**
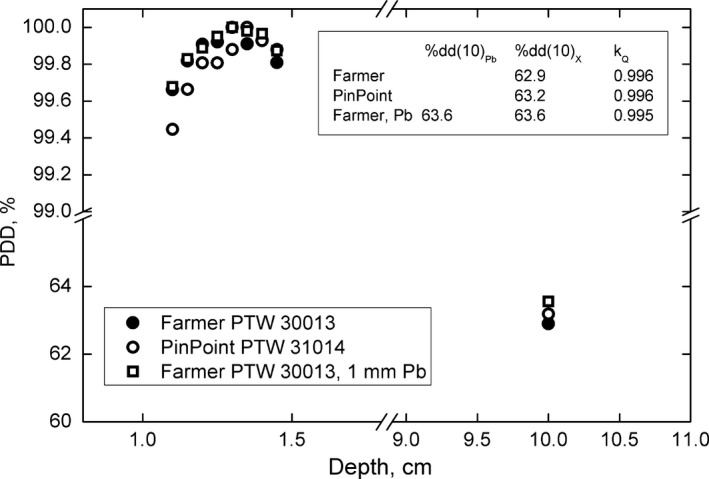
Percent depth dose measurements performed with Farmer and PinPoint chambers. Farmer data is presented for lead filtered and unfiltered beams.

Percent depth dose measurements performed in water with the Farmer and PinPoint chambers are shown in Fig. 3. Measurements were performed for a 10 × 10 cm^2^ field, defined by the MLC, delivered at SSD = 100 cm. Three PDD measurements were performed: (a) with a Farmer chamber and lead filtering 30 cm from the water surface, (b) with a Farmer chamber, no lead filtering, and (c) with a PinPoint chamber, no lead filtering. Because the value of %dd(10)_Pb_ measured with the Farmer chamber was less than 71%, we used the provision that %dd(10)_x_ = %dd(10)_Pb_, as outlined in the original TG‐51 report.^1^ Thus, measured values of %dd(10)_X_ were 63.6%, 62.9%, and 63.7% for the measurements described above, respectively.

Fitting parameters specified for the Farmer chamber (Table 1 of the TG‐51 addendum) were used to calculate values of *k*_Q_. PinPoint chambers are not recommended for absolute calibration,^4^, ^5^ and use of this chamber was limited to relative dosimetry. The calculated values of *k*_Q_ were 0.995, 0.996, and 0.996 for the three measurements, respectively. The value of *k*_Q_ calculated using the lead‐filtered, Farmer‐measured PDD, 0.995, was used for reference dosimetry.

**Table 1:** *P*_ion_ and *P*_pol_ for PTW 30013 Farmer chamber in Halcyon™, TrueBeam^®^ and Clinac^®^ beams

	Halcyon™ 6 MV‐FFF	TrueBeam^®^ 6 MV‐FFF^a^	TrueBeam^®^/Clinac^®^ 6 MV^b^
*P*_ion_	1.0066	1.0066 ± 0.0003	1.0031 ± 0.0004
*P*_pol_	0.9994	0.9994 ± 0.0001	0.9994 ± 0.0002

Uncertainties are standard deviations.

a TrueBeam^®^ 6 MV‐FFF data averaged over four annual calibrations.

b 6 MV data averaged over four matched machines (TrueBeam^®^ and Clinac^®^) and four annual calibrations.

### 3.B Reference dosimetry

**Figure 4:**
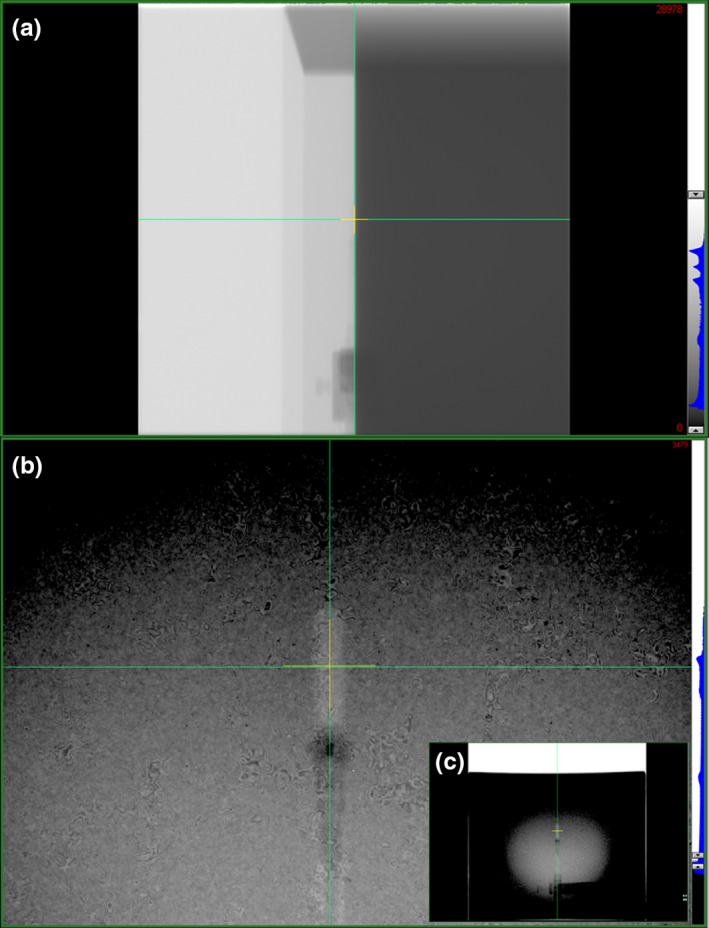
MV images acquired at gantry angles of 90° (a) and 0° (b, c) for alignment of water tank and Farmer chamber at isocenter. Green crosshairs indicate isocenter while yellow crosshairs indicate the center of the imager.

Examples of the orthogonal MV images used for chamber positioning are shown in Fig. 4. Based on imaging, the chamber position was adjusted with lateral and vertical couch shifts. All shifts were less than 2 mm in magnitude.

Values of *P*_ion_ and *P*_pol_ were determined as described by the TG‐51 protocol, by recording the charge produced in the Farmer chamber at 10 cm depth for a 10 × 10 cm^2^ field delivered at SSD = 100 cm using electrometer biases of −300, +150, and +300 V. Three readings at each polarity were obtained and averaged to determine *P*_ion_, *P*_pol_, and *M*_raw_. The values of *P*_ion_ and *P*_pol_ for the Halcyon™ are compared against those determined for a Varian TrueBeam^®^ linear accelerator (6 MV‐FFF and 6 MV beams) and for a Varian Clinac^®^ linear accelerator (6 MV only) in Table 1. *P*_ion_ differs between the filtered and unfiltered beams by 0.0035 while *P*_pol_ matches for both beams.

*P*_rp_ was estimated according to the TG‐51 addendum to be 1.003 at *d*_max_ and 1.0006 at 10 cm depth. This resulted in an overall correction of 1.002 when %dd(10) was applied to relate measured output at 10 cm depth to output at *d*_max_.

## 4 DISCUSSION

In the absence of a front‐pointer positioning mechanism and a light field, MV imaging was successfully employed to setup a water tank and Farmer chamber for TG‐51 reference dosimetry of a Halcyon™ linear accelerator. Orthogonal MV images demonstrated good initial positioning of the chamber using the externally mounted bore lasers, requiring couch shifts less than 2 mm in the vertical and lateral directions. The vertical shift accounts for sag in the couch due to the weight of the water tank, while the lateral shift is a systematic one identified for our machine's calibration of the virtual‐to‐actual isocenter shift. No longitudinal adjustments were required.

Following installation of the Halcyon, and before any output tuning was performed, the output of the machine was measured to be 1.0318 cGy/MU under the factory default reference point definition (*d*_max_ for 100 cm SSD). After performing reference dosimetry as described above, the output of the machine was measured to be 0.9993 cGy/MU and independently verified by the IROC Houston Quality Assurance Center, using OSLDs, to be 0.997 cGy/MU. Despite differences in the 6 MV‐FFF beams produced by the Halcyon™ and TrueBeam^®^ accelerators, measured values of *P*_ion_ and *P*_pol_ were the same when TrueBeam data was averaged over four years.

The TG‐51 addendum is clear in its recommendation to use lead filtering and, consequently, to employ eqs. (13) and (14) for the determination of %dd(10) for FFF beams at all energies. However, there is no explicit discussion of the difference, if any, in electron contamination down‐stream of the lead between flattened and unflattened beams. In the paper by Rogers^6^ establishing eqs. (13) and (14) for use in the TG‐51 protocol, it is recommended that for %dd(10)_Pb_ < 71% or 73%, depending on the position of the lead, the measurement can be performed without the lead given that electron contamination has a negligible effect on %dd(10) at these energies. For FFF beams, we know this is not the case.^7^ Given the successful implementation of TG‐51 reference dosimetry for unflattened beams used in clinics throughout the world for over a decade, the impact of this ambiguity appears minimal, however, this is a matter that would benefit from some clarity.

Ultimately, the Halycon™ was designed to require little maintenance for clinics with limited access to on‐site Varian support, and streamlined in terms of setup and use for clinics with limited access to medical physics training or staff. Any simplification of the reference dosimetry procedure would provide a safety advantage for clinics of the later description. The TG‐51 and addendum reports^1^, ^4^ recommend the use of lead‐filtered PDDs for the specification of beam quality and *k*_Q_ for FFF beams, however, values of %dd(10)x for the Halcyon™ 6 MV‐FFF beam determined using a Farmer chamber with and without filtering differ by 0.7% resulting in *k*_Q_ values of 0.995 and 0.996. This represents a 0.1% difference in output if lead filtering were omitted from the reference dosimetry of this machine, simplifying the reference dosimetry process with limited impact on accuracy. While lead filtering should still be used whenever possible to comply with the addendum, its omission is ultimately preferable to incorrect use.

Additionally, the commissioning of flattening filter free fields is often accompanied by complications related to the dosimetry of such forward‐peaked beams. The broadened profile of the Halcyon™ 6 MV‐FFF beam means that issues related to dose averaging over the active volume of typical cylindrical chambers positioned along the central axis are also greatly reduced. PDDs acquired with the Farmer and PinPoint chambers are very similar, and differ by only 0.3% at 10 cm depth in water. This indicates that a clinic with limited dosimetry tools should be able to accurately commission the Halcyon beam for clinical use with only a Farmer chamber.

During couch loading, it was observed that the fixed speed of couch resulted in substantial agitation of the water within the water tank. Beyond requiring tens of minutes to still the water, we found that an over‐full water tank may spill onto the couch and gantry cover below, therefore, we recommend the water tank not be overfilled and that towels be kept close at hand during couch loading to quickly attend to spills.

## 5 CONCLUSION

TG‐51 reference dosimetry was successfully implemented on the Halcyon™ linear accelerator using orthogonal MV imaging to accurately position the ion chamber at the radiation isocenter. Despite profile differences in the 6 MV‐FFF beams produced by the Halcyon™ and TrueBeam^®^ accelerators, TG‐51 correction factors were the same for both machines, while Halcyon's broad profile means that Farmer chambers can be used for PDD acquisition without concerns of volume averaging. Additionally, values of *k*_Q_ calculated from PDDs acquired with and without lead filtering differed by 0.001, corresponding to differences in output of only 0.1%.

## CONFLICT OF INTEREST

The authors have no conflicts of interest to declare.
